# Reduction of T Cell Receptor Diversity in NOD Mice Prevents Development of Type 1 Diabetes but Not Sjögren’s Syndrome

**DOI:** 10.1371/journal.pone.0112467

**Published:** 2014-11-07

**Authors:** Joanna Kern, Robert Drutel, Silvia Leanhart, Marek Bogacz, Rafal Pacholczyk

**Affiliations:** Center for Biotechnology and Genomic Medicine, Georgia Regents University, Augusta, Georgia, United States of America; National Institute of Dental and Craniofacial Research, United States of America

## Abstract

Non-obese diabetic (NOD) mice are well-established models of independently developing spontaneous autoimmune diseases, Sjögren’s syndrome (SS) and type 1 diabetes (T1D). The key determining factor for T1D is the strong association with particular MHCII molecule and recognition by diabetogenic T cell receptor (TCR) of an insulin peptide presented in the context of I-A^g7^ molecule. For SS the association with MHCII polymorphism is weaker and TCR diversity involved in the onset of the autoimmune phase of SS remains poorly understood. To compare the impact of TCR diversity reduction on the development of both diseases we generated two lines of TCR transgenic NOD mice. One line expresses transgenic TCRβ chain originated from a pathogenically irrelevant TCR, and the second line additionally expresses transgenic TCRα^mini^ locus. Analysis of TCR sequences on NOD background reveals lower TCR diversity on Treg cells not only in the thymus, but also in the periphery. This reduction in diversity does not affect conventional CD4^+^ T cells, as compared to the TCR^mini^ repertoire on B6 background. Interestingly, neither transgenic TCRβ nor TCR^mini^ mice develop diabetes, which we show is due to lack of insulin B:9–23 specific T cells in the periphery. Conversely SS develops in both lines, with full glandular infiltration, production of autoantibodies and hyposalivation. It shows that SS development is not as sensitive to limited availability of TCR specificities as T1D, which suggests wider range of possible TCR/peptide/MHC interactions driving autoimmunity in SS.

## Introduction

NOD mice serve as well-established models of independently developing autoimmune diseases, Type 1 Diabetes (T1D) and Sjögren’s syndrome (SS) [1], [2]. T1D is characterized by autoimmune attacks against the pancreatic beta-cells with T cells playing an essential role in the initiation and progression of the disease, leading to hyperglycemia and vascular complications [3], [4]. SS is an autoimmune disease with local and systemic manifestations, characterized by mononuclear infiltrates into salivary and lacrimal glands leading to clinical symptoms of dry mouth and dry eyes [5], [6]. Glandular infiltrates consist mostly of CD4^+^ T cells with lesser amounts of CD8^+^ T cells and B cells. Although factors like viral or bacterial infections, aberrant glandular development or cytokine production are important in the initial phase of the pathogenesis of SS, CD4^+^ T cells are important players in the onset of autoimmunity and disease progression.

Autoimmunity in NOD mice is attributed to several different events occurring in the thymus and in the periphery. Studies in these mice showed a defect in negative selection [7], perturbed αβ/γδ lineage decision leading to a shift in selection niches [8], reduced relative diversity of thymic Treg cells [9], peripheral hyper-responsiveness of effector CD4^+^ T cells [10], multiple binding registers of insulin B:9–23 peptide resulting in poor negative selection in the thymus [11], [12], or peripheral post-translational modification of self-peptides/neo-antigens [13]. Despite genetic predispositions, the key component in the development of autoimmune diseases is the recognition of a particular antigen in the context of MHC Class II molecule by CD4^+^ T cells. The development of diabetes in NOD mice is associated with the key I-A^g7^ molecule (HLA-DQ8 in humans) in the absence of a functional I-E molecule [14], [15]. Co-expression of other MHC molecules with I-A^g7^ can prevent development of diabetes in a dominant fashion [14], [15]. Replacement of I-A^g7^ with other MHC molecules, like I-A^b^, I-A^p^ or I-A^q^, does not promote the development of diabetes yet mice continue to develop autoimmune exocrinopathy and the severity of the SS and the profile of antibodies’ specificities vary between congenic mice [16]. In large-scale association study of SS in humans, HLA was found to have the strongest linkage to the disease [17].

The strict dependence of T1D on the particular MHC allele correlates with its primary antigen requirement where insulin B:9–23 peptide has been identified as the epitope necessary for onset of the disease in NOD mice [18]. In SS, no key epitope(s) are identified, although several proteins have been implicated as a source of antigens: Ro/SSA 52 kDa, αFodrin, Muscarinic Acetylcholine 3 Receptor (M3R), α-amylase, islet cell autoantigen-69, kallikrein-13 [19]–[24]. Recently it has been shown that the transfer of T cells from M3R-immunized M3R^−/−^ mice into Rag^−/−^ mice leads to development of sialadenitis, showing pathogenic potential of M3R specific T cells [25].

Despite the strict requirement of the presence of the insulin B:9–23/I-A^g7^ combination, the development of T1D in NOD mice proceeds even when total TCR diversity and precursor frequency of diabetogenic TCRs is limited. The reduction of TCR diversity by use of TCRβ transgenic mice [26], or great reduction of precursor frequency relying on allelic exclusion escapees on NOD background does not prevent development of T1D [27], although not all endogenous TCRβ chains are permissive for the development of insulin B:9–23 specific TCRs [28]. In SS it is not clear as to what role diversity of interactions between TCRs and different peptide/MHCII complexes play in the onset and development of the disease. Previous studies in patients with SS found that TCR repertoire of infiltrating T cells is to some extent restricted with different dominant clonotypes of Vβ families [29]–[33]. Despite the lack of dominant Vα/Vβ families or dominant specificity in different patients these studies show clonal expansion of infiltrating T cells, which suggests that the number of epitopes participating in the autoimmunity of the disease is limited [34], [35]. However, the weaker dependence of SS on MHC polymorphism suggests broad diversity of possible TCR/peptide/MHCII interactions participating in the pathogenesis of the disease. As the diversity of antigenic specificities and TCR repertoire on T cells involved in SS development is not well understood, we wanted to compare sensitivity of development of SS versus T1D to the diminishing diversities of TCRs in the presence of the same I-A^g7^ molecule in NOD mice. To reduce the diversity of TCRs in NOD mice we generated two types of transgenic strains, in which all T cells express either one transgenic TCRβ chain or additionally co-express TCRα chains from TCRα^mini^ locus [36]. Interestingly, these mice do not develop T1D but still develop SS with glandular infiltrations, autoantibody presence and hyposalivation. We investigated the reasons for the lack of T1D development and the role of reduced diversity on generated repertoires of TCRs on conventional and regulatory T cells in the thymus and periphery of NOD mice.

## Materials and Methods

### Ethics statement

All mice used in this study were housed in the animal care facility at the Georgia Regents University (GRU). All work involving animals was conducted under protocols approved by the Animal Care and Use Committee at the GRU (#2008−0231). All efforts were made to minimize suffering. Mice were euthanized by CO2 followed by cervical dislocation.

### Mice

Production of TCRβ^Tg^ and TCRα^mini^ constructs and generation of transgenic mice on C57BL/6 (B6) background was described previously (Pacholczyk 2006). A similar strategy was used to microinject one or both DNA constructs into zygotes of NOD mice (Transgenic Mice Core Facility, GRU). To eliminate expression of endogenous TCRα chains, NOD.TCRβ^Tg^.TCRα^mini^ transgenic mice were crossed with NOD.TCRα^−/−^ (NOD.129P2(C)-Tcratm1Mjo/DoiJ) mice purchased from The Jackson Laboratory (Bar Harbor, ME). To facilitate identification of Treg cells both transgenic lines then were crossed with NOD.FoxP3GFP/cre mice (NOD/ShiLt-Tg(Foxp3-EGFP/cre)1Jbs/J) purchased from The Jackson Laboratory. The B6.Aec1Aec2 (B6.DC) mice were kindly provided by Dr. Ammon Peck [37].

### Histology

Organs were removed from each mouse at the time of euthanasia, placed in 10% phosphate-buffered formalin for at least 24 h and then embedded in paraffin. Sections were taken at 5 µm of thickness 200 µm apart. The tissue sections were stained with hematoxylin and eosin (H&E) at the Histology Core Laboratory, GRU. One infiltrate was defined as a cluster of at least 50 nucleated cells, scoring described in figures.

### Measurement of saliva flow

Mice were given an i.p. injection of 100 µl of pilocarpine (0.05 mg/ml in PBS) per 20 g of body weight. Saliva was collected for 10 min., starting 1 min. after injection of pilocarpine. The volume of saliva was measured and normalized to the mouse body weight.

### Detection of auto-antibodies

Auto-antibodies and total IgG1 were measured using mouse serum with the following ELISA kits: αFodrin (American Research Products), ANA, ssDNA, dsDNA (Immuno-Biological Laboratories) and IgG1 (Immunology Consultants Laboratory). Assays were performed according to manufacturer’s protocols with serum dilution 1∶100 and 1∶50,000 for IgG1. OD_450_ values of negative samples were subtracted from the OD_450_ of experimental samples.

### Cell preparation, flow cytometry and cell sorting

Cells were isolated from peripheral lymph nodes (axillary, brachial and inguinal) and thymii by mechanical disruption through nylon mesh. Salivary mandibular and extraorbital lacrimal glands were first cut and digested using collagenase (1 ug/ml) for 30 min in 37°C. Cells were washed and counted (Countess, Invitrogen) and used for staining with monoclonal antibodies: CD4 (clone RM4-5), CD8α (53-6.7), B220 (RA3-6B2), TCRVα2 (B20.1), TCRVβ14 (14-2), TCRβ (H57-597), CD25 (PC61), CD45RB (16A), CD62L (MEL-14), all from BD Biosciences. Stained cells were either analyzed using FACS Canto (BD Biosciences) or sorted on MoFlo Sorter (Cytomation). Dead cells were excluded using forward vs side scatter dot plots and doublets discrimination was accomplished using forward scatter height vs width dot plots. Purities of all sorted populations were above 98%.

### Immunization and generation of T cell hybridomas

Mice were immunized at the base of the tail with 50 µg of insulin B:9–23 peptide emulsified in Complete Freund’s Adjuvant (Thermo Scientific). One week later lymphocytes were isolated from draining lymph nodes and cultured *in vitro* for 3–4 days with insulin B:9–23 peptide (50 µg/ml), followed by 3–4 days of expansion with murine recombinant IL-2 (20 U/ml, Peprotech). For generation of allo-specific T cell hybridomas, CD4^+^ T cells sorted from un-immunized experimental mice were stimulated *in vitro* by co-culture with splenocytes from B6.TCRα^−/−^ mice, followed by expansion with IL-2. Activated T cells were fused to BW5147 TCRα-β- NFAT-EGFP cells as previously described [38]. BW NFAT-EGFP fusion partner expresses GFP protein under the minimal human IL-2 promoter, which contains NFAT-binding sites [39]. T cell hybridomas were selected using HAT (Cellgro) selection media by limiting dilution method. For stimulation, cloned T cell hybridomas (10^5^ cells) were co-cultured overnight with 5×10^5^ splenocytes (from NOD.TCRα−/− or B6.TCRα−/− mice) with or without insulin B:9–23 peptide (50 µg/ml) or anti-CD3 antibody (1 µg/ml). Specific activation of the hybridomas was measured by detection of GFP-positive cells or by detection of IL-2 in culture supernatant using CBA Mouse IL-2 Flex Set (BD Biosciences).

### MTT assay

The proliferation of the cells was measured using 3-(4,5-dimethythiazol-2-yl)-2,5-diphenyltetrazolium bromide (MTT; Sigma) assay. On the third day of *in vitro* stimulation 10 µl of MTT (5 mg/ml) was added to each well and incubated for 4 h. After discarding supernatant, the remaining formazan precipitates were dissolved in 150 ul of 70% isopropanol solution (70% isopropanol, 30% water, 0.02N hydrochloric acid) overnight. Absorbance was measured at 570 nm using Microplate Reader (Biotek Synergy HT).

### Sequencing

Single cell sorting and sequencing was done as previously described [36]. High-throughput sequencing was done using Ion Torrent platform (Life Technologies) by Genomic Core Facility at GRU. Libraries of the TCRs were prepared according to protocol. Shortly, CDR3α regions were amplified using primers specific for Vα2 and Cα segments with integrated adapters and barcodes, provided by manufacturer. Before sequencing, consistency of samples was checked by 2D-F-SSCP analysis of PCR products from three aliquots of cDNA per each sample [36]. Only samples with three similar/identical profiles were considered without PCR bias and were used for further purification using Agencourt AMPure XP reagent (Beckman Coulter) and used for sequencing. FASTQ files with sequences were processed and analyzed using custom-written program in Pearl (ActivePearl, ActiveState Software Inc.). Sequences with quality score of CDR3α region above 27 were used for analysis. The length of CDR3α region was defined by counting from the third amino acid after the invariant C residue in all Vα regions (Y-L/F-C-A-X-first) to the amino acid immediately preceding common Jα motif (last-F-G-X-F-G-T).

### Statistical analysis

The similarity, diversity and richness estimators were calculated using programs designed to measure biodiversity: EstimateS8.2 (Colwell, R. EstimateS: Biodiversity Estimation Software. Program and User’s Guide at http://viceroy.eeb.uconn.edu/estimates) and SPADE (Chao, A. and Shen, T.-J. (2010) Program SPADE (Species Prediction And Diversity Estimation). Program and User’s Guide published at http://chao.stat.nthu.edu.tw).

## Results

### Development of T cells in NOD^βTg^ and NOD^mini^ mice

We generated TCRβ transgenic mice, in which all T cells use the same TCRβ^Tg^ chain (Vβ14Dβ2Jβ2.6) and one of the endogenous TCRα chains. The TCRβ transgenic chain originated from I-A^b^ restricted TCR specific to Ep63 K peptide an analog of Eα 52–68 peptide in which the residue at position 63(I) was substituted with lysine [40]. To further reduce diversity of TCRs we used TCRα^mini^ transgenic construct, used previously to create TCR^mini^ mouse on B6 background (B6^mini^) [36]. The TCRα^mini^ transgene allows a single Vα2.9 segment to rearrange to one of the two Jα (Jα26 and Jα2) segments. The generated transgenic mice were further crossed with TCRα^−/−^ mice to ensure that all developing T cells use transgenic TCRα^mini^ locus. To track CD4^+^ Foxp3^+^ regulatory T (Treg) cells we crossed transgenic mice with NOD.Foxp3.EGFP/cre mice as described in methods. Final characteristics of mice used in this paper were NOD^mini^ (NOD.TCRα^mini^.TCRβ^Tg^.TCRα^−/−^.Foxp3EGFP/cre), NOD^βTg^ (TCRβ^Tg^.TCRα^+/−^.Foxp3EGFP/cre) and NOD (NOD.Foxp3EGFP/cre).

**Figure 1:**
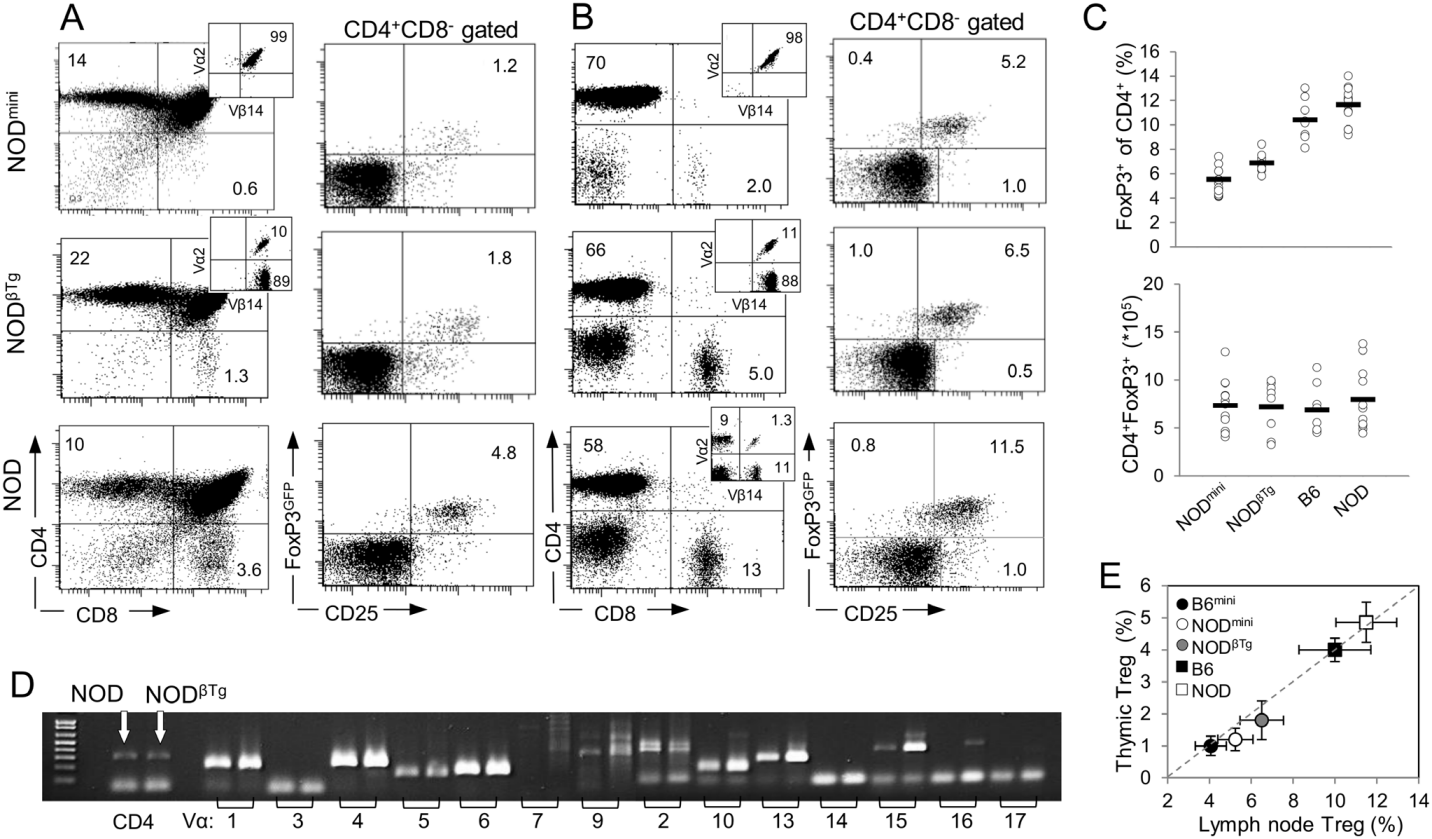
Efficient selection of CD4^+^ T lymphocytes in NOD^mini^ and NOD^βTg^ mice. Lymphocytes isolated from thymii (A) and lymph nodes (B) of indicated mice were stained with monoclonal antibodies and analyzed by flow cytometry. Numbers in quadrants are representative percentages of at least six mice (6 week old) per group. The numbers of thymocytes and lymphocytes ± SD recovered from NOD^mini^, NOD^βTg^ and NOD mice were: from thymii 78.5±16.7×10^6^, 72.05±13.8×10^6^ and 70.1±14.0×10^6^, and from lymph nodes (axillary, brachial and inguinal) 17.2±5.8×10^6^, 14.1±2.5×10^6^, and 13.2±3.0×10^6^, respectively. (C) Percentages (top) and total numbers (bottom) of CD4^+^Foxp3^+^ T cells in peripheral lymph nodes of 6 week old mice; each circle represents individual mouse. (D) Expression of mRNA of TCRVα genes in sorted CD4^+^ T cells isolated from NOD and NOD^βTg^ mice. Analysis was done by RT-PCR using primers specific to indicated Vα segments and Cα region. (E) Comparison of CD4^+^Foxp3^+^ T (Treg) cells from thymii and peripheral lymph nodes of transgenic and wild type B6 and NOD mice. Mean percentage and SD of six young mice per group are shown.

In NOD^mini^ and NOD^βTg^ transgenic mice thymic development of T cells proceeds normally, and selection of single positive (SP) thymocytes is very efficient with bias toward CD4^+^ T cells in the thymus and in the periphery, similarly to a previously reported bias on B6 background (Fig. 1A, 1B) [36]. TCRβ transgenic mice are characterized by allelic exclusion, resulting in all T cells to express only one transgenic TCRβ chain. It has been proposed that allelic exclusion in NOD mice is less efficient [27]. In our mice all SP thymocytes and peripheral T cells have exclusive expression of transgenic TCRβ^Tg^ chain (TCRVβ14) in both types of transgenic mice and exclusive expression of TCRα^mini^ transgene (TCRVα2) in NOD^mini^ mice (Fig. 1A, 1B). Also we did not observe emergence of the cells bearing TCRs with Vβ segments other than Vβ14 even in older mice. Furthermore, we observed lower percentages of Treg cells in thymii and periphery of transgenic mice as compared to NOD mice and the percentages correlated with diversities of TCRs in analyzed mice (Fig. 1C, 1E). Nevertheless, the total numbers of peripheral Treg cells were similar in all of analyzed mice and reduced percentages of Treg cells were due to more efficient selection of CD4^+^ T cells, rather than diversity of TCRs, as we observed this correlation on B6 background and in other types of mice that differ by efficiency of CD4^+^ T cells selection (Fig. 1C, 1E) [36], [41]. Finally, to check whether this particular TCRVβ14 transgenic chain can impose an unusual restriction on the TCRα repertoire, we evaluated the frequency of individual Vα families. We found no bias in TCR repertoire as the frequency of Vα families usage by CD4^+^Vβ14^+^ T cells was similar between NOD and NOD^BTg^ mice (Fig. 1D).

### Lack of T cells specific to insulin B:9–23 in NOD transgenic mice

**Figure 2:**
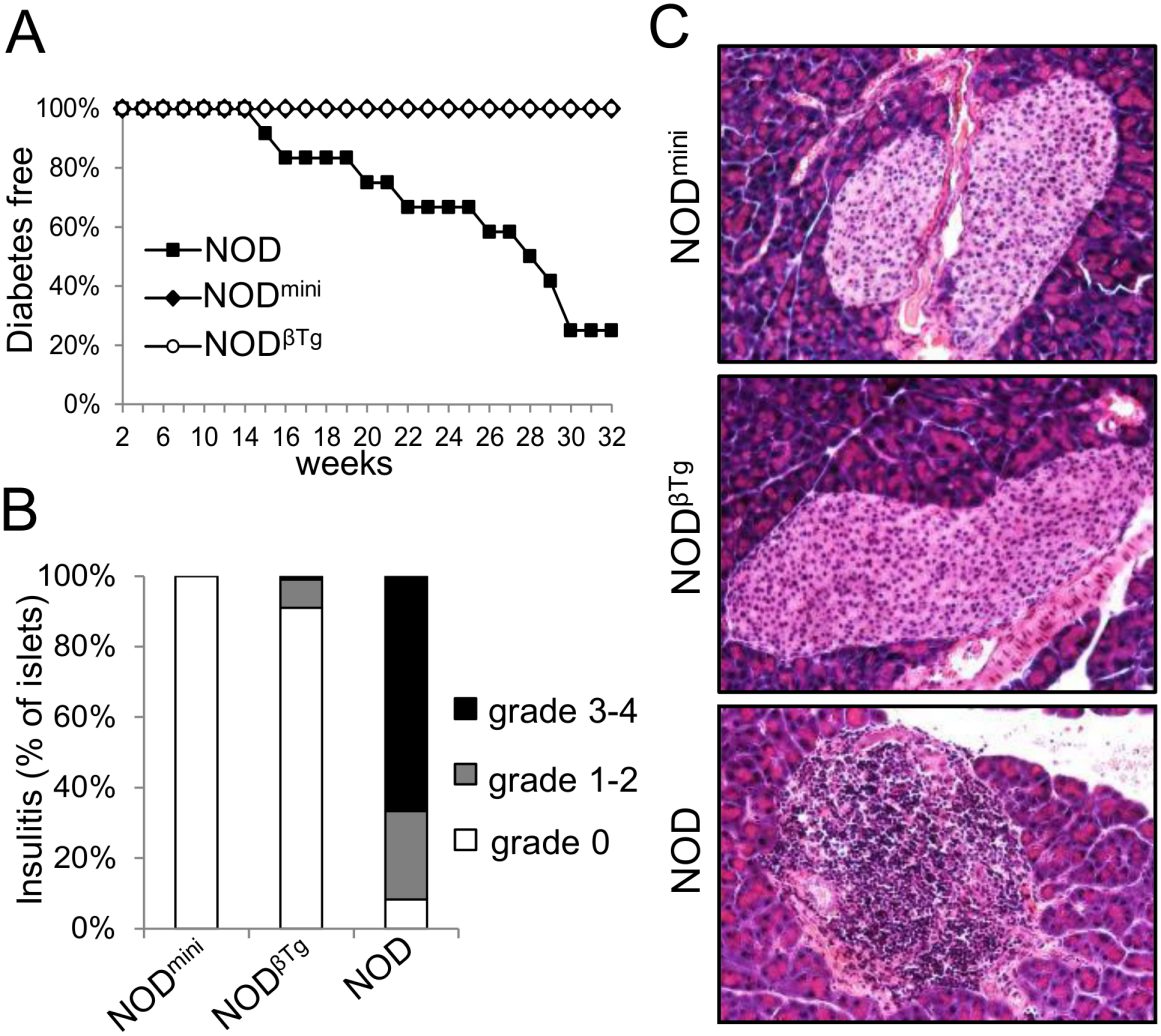
NOD^βTg^ and NOD^mini^ mice do not develop T1D. (A) Incidence of diabetes in mice shown as Kaplan–Meier survival curve. Mice with three consecutive measurements of blood glucose level above 250 mg/dL were considered diabetic. At least twelve mice per group were analyzed; p<0.05. (B) H&E staining of pancreatic tissue sections were analyzed for lymphocytic infiltrates and percentages of islets with grade of insulitis were counted. Insulitis was graded based on following criteria: no infiltrates – grade 0; peri-insulitis - grade 1; insulitis <25% - grade 2; insulitis <50% - grade 3; insulitis >50% - grade 4. At least six mice at 20 weeks of age were analyzed with the total number of 140 (NOD^mini^), 155 (NOD^βTg^) and 160 (NOD) islets. (C) Example of H&E staining of pancreatic tissue sections of indicated mice at 20 weeks of age.

To test development of diabetes we measured blood glucose levels in experimental mice. Surprisingly, neither NOD^βTg^ nor NOD^mini^ transgenic mice develop diabetes (Fig. 2A). Evaluation of H&E stained pancreatic sections showed lack of lymphocytic infiltrates in 25 week old transgenic mice, with only insignificant infiltrates found in a few sections of NOD^βTg^ mice (Fig. 2B). As a control we used non-transgenic littermates of NOD^βTg^, NOD.TCRα^+/−^ mice. These mice developed diabetes and by 30 weeks of age more than 75% of females had high levels of blood glucose (Fig. 2A). One of the possibilities was that the lack of development of diabetes in transgenic mice is due to perturbed proportions of regulatory to effector T cell ratios, which could result in more efficient suppression of potentially diabetogenic T cells. We took advantage of cyclophosphamide treatment, which selectively affects numbers and function of Treg cells and accelerates development of diabetes in NOD mice [42], [43]. This treatment however did not cause insulitis nor diabetes in transgenic mice indicating inability of effector T cells to initiate autoimmunity (unpublished data).

**Figure 3:**
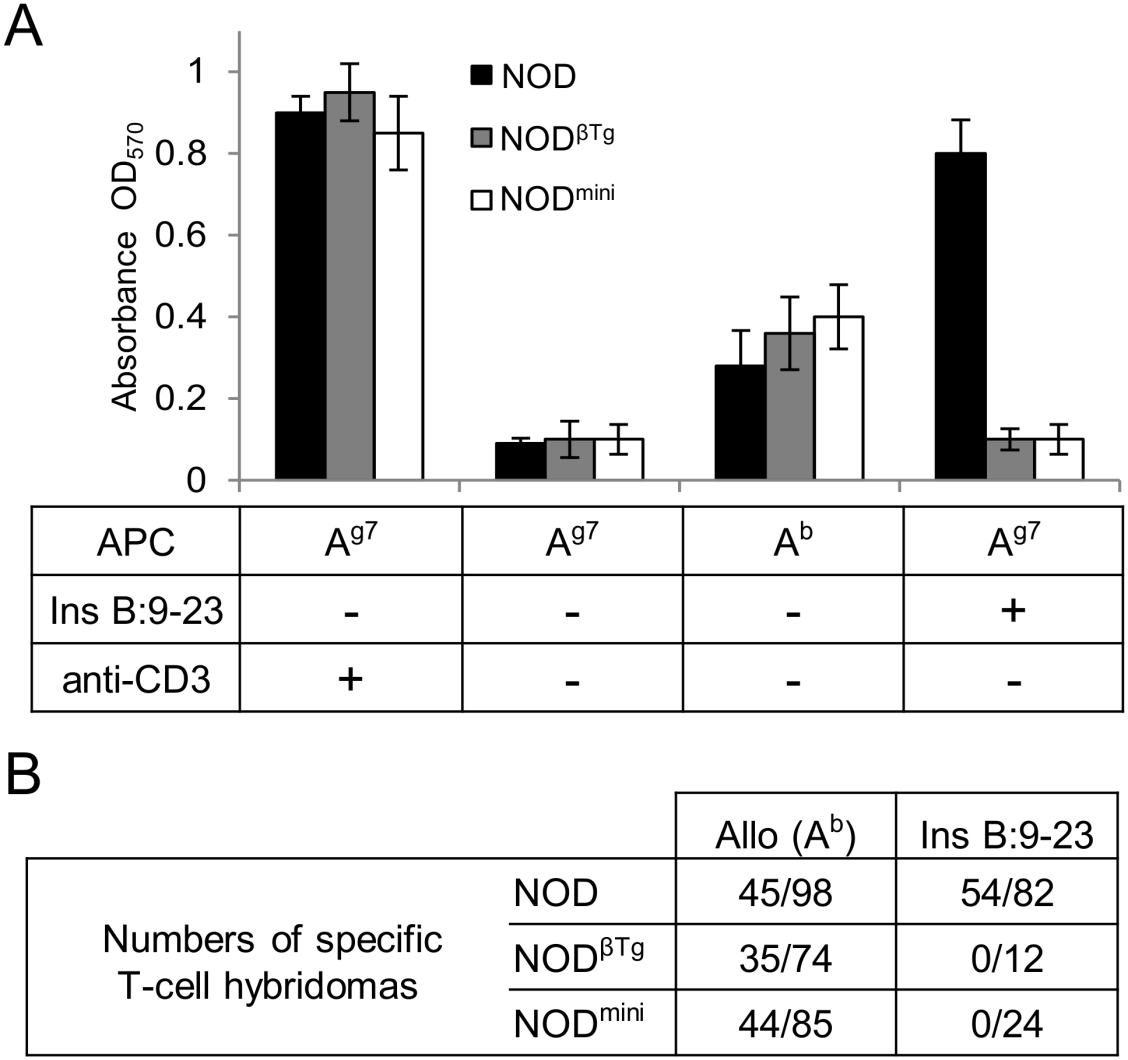
Lack of response to insulin B:9–23 peptide in transgenic NOD mice. (A) 5×10^4^ of CD4^+^ T cells sorted from lymph nodes of NOD, NOD^βTg^ and NOD^mini^ mice were cultured in the presence of 5×10^5^ splenocytes from NOD.TCRα^−/−^ (A^g7^) or B6.TCRα^−/−^ (A^b^) mice and soluble anti-CD3 (1 µg/ml) or insulin B:9–23 peptide (50 µg/ml), as indicated. Proliferation of cells was measured after 3 days by MTT assay [38]. Experiments were done twice with 3 mice per group. (B) T-cell hybridomas specific to allo-antigens or insulin B:9–23 peptide were generated from indicated mice. For generation of B:9–23 specific hybridomas, mice were immunized with the peptide 7 days prior to isolation of lymph nodes for *in vitro* blasts generation [38]. Generated hybridomas were tested for their ability to respond to syngeneic (NOD.TCRα^−/−^) or allogeneic (B6.TCRα^−/−^) splenocytes with or without B:9–23 peptide or anti-CD3. Table shows numbers of identified hybridomas specific to indicated antigens and numbers of hybridomas responding to anti-CD3 stimulation. Table is representative of 3 independent experiments with two mice per group.

The onset of spontaneous diabetes in NOD mice is dependent upon presence of effector T cells specific to insulin B:9–23 antigen and lack of such specificity results in lack of early infiltrates into pancreatic islets [18]. As our mice did not develop islet-infiltrates, we tested their ability to respond to stimulation by insulin B:9–23 peptide (Fig. 3). CD4^+^ T cells isolated from NOD^mini^, NOD^βTg^ and NOD mice responded to stimulation by allogeneic splenocytes, however only CD4^+^ T cells from immunized NOD mice were able to respond to B:9–23 peptide in the presence of syngeneic splenocytes (Fig. 3). As of note, B:9–23 peptide does not bind to I-A^b^ molecule and in the presence of B6 splenocytes does not lead to CD4 T cell response [44]. We were also unable to generate B:9–23 specific T cell hybridomas from immunized NOD^mini^ and NOD^βTg^ mice, however we had no problem generating allo-specific T cell hybridomas from transgenic mice. These results showed that changes introduced by transgenic chains to the TCR repertoire in NOD mice resulted in lack of peripheral specificity to the key antigen required for onset of T1D.

### Development of SS in NOD^βTg^ and NOD^mini^ mice

**Figure 4:**
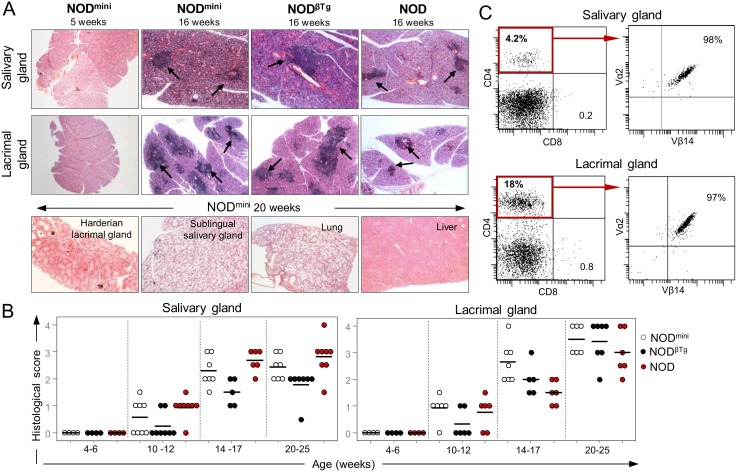
Lymphocytic infiltrates in mandibular salivary and extraorbital lacrimal glands. (A) H&E staining of tissue sections from indicated organs of analyzed mice, showing lymphocytic infiltrates indicated by arrows. (B) Histological score of infiltrated glands in indicated age groups. Scoring criteria: score 0, no infiltrates; score 1–1.5, 1–2 foci per section; 2–2.5, 3–5 foci per section; score 3, 6–10 foci per section; score 4, more than 10 foci per section. Infiltrate is considered as focus when number of infiltrating cells in continuous space is greater than 50. Three sections at different anatomical locations per organ were analyzed with at least 5 mice per age group. (C) FACS analysis of CD4 T cells infiltrating into salivary and lacrimal glands in 16 week old NOD^mini^ mice. Dot plots on the right show expression of transgenic TCR on CD4^+^ gated cells.

The lack of T1D in transgenic mice prompted us to test whether reduced TCR diversity will affect development of Sjögren’s syndrome – secondary autoimmune disease in NOD mice. It is characterized by lymphocytic infiltrates into salivary and lacrimal glands, production of auto-antibodies and the loss of saliva and tear production by 20 weeks of age [5], [45]. Histological evaluation of tissue sections of submandibular salivary and extraorbital lacrimal glands showed focal lymphocytic infiltrates, in both transgenic NOD^mini^ and NOD^βTg^ and parental NOD mice (Fig. 4). Similarly to the NOD mice, males of both transgenic mice had milder infiltration of salivary glands than females, while the infiltration of lacrimal glands was more prominent in male NOD^βTg^ and NOD^mini^ mice. This infiltration is organ specific, as we did not detect infiltrates in lungs, kidney, liver, as well as sublingual and parotid salivary glands and harderian lacrimal glands of 20 week old transgenic mice, similarly to NOD mice (Fig. 4B). Of note, in some of 30 week old NOD^mini^ females, but not NOD^βTg^ mice, we noticed development of lymphoproliferation, manifested by enlarged lymph nodes and spleen and infiltration into multiple organs (unpublished data). FACS analysis of lymphocytic infiltrates in affected exocrine glands showed that all of the infiltrating CD4^+^ T cells expressed transgenic Vβ14 chain in NOD^βTg^ mice and Vβ14/Vα2 transgenic chains in NOD^mini^ mice (Fig. 4C). This also showed the aforementioned stability of expression of transgenic TCRs in experimental mice.

**Figure 5:**
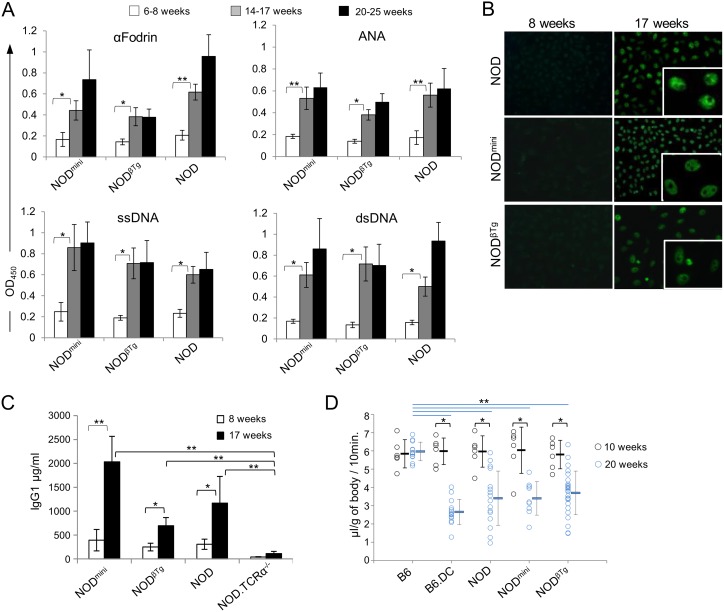
Detection of autoantibodies and hyposalivation in NOD^mini^ and NOD^βTg^ mice. (A) ELISA assay was performed using mouse serum (1∶100) from indicated age groups. Each graph represents mean value of OD_450_ and standard deviation for indicated antigens. At least six mice were used per each group. *p<0.05, **p<0.005. (B) Detection of ANAs pattern using Hep-2 cell line. Sera from mice were diluted 1∶40, incubated with HEp-2-fixed slides and evaluated under fluorescent microscope at x20 magnifications. Representative images are shown. (C) Quantitative ELISA analysis of IgG1 levels in sera (1∶50,000) from indicated mice. Each bar represents mean value and standard deviation from six mice per experimental groups. (D) Salivary flow rates after pilocarpine injection in indicated mice at 10 and 20 wks of age. Double congenic B6.NODIdd3.NODIdd5 (B6.DC) mice, that develop SS on B6 genetic background, were used as a control. Saliva volume was measured and calculated in mg per mouse body mass. Each circle represents one mouse and horizontal lines indicate mean values of the experimental groups. T-test was used to calculate differences between groups. *p<0.002, **p<0.0002.

Development of SS in humans is often correlated with presence of anti-nuclear antibodies (ANA), anti-Ro/SSA, anti-La/SSB, anti-dsDNA, anti-αFodrin and anti-M3R [5], [22], [46]. In the NOD mouse model of SS, anti-SSA and anti-SSB auto-antibodies are rarely present and they are found at the very low levels [47]. To determine the presence of auto-antibodies in experimental mice, we used ELISA-based assay. For negative control, we used sera from NOD.TCRα^−/−^ mice, that do not have T cells. As shown in Fig. 5, transgenic and parental NOD mice from 14–17 week old group had elevated levels of antibodies against αFodrin, ssDNA, dsDNA and ANA. Additionally, we determined the staining pattern of ANA auto-antibodies using immunofluorescent staining of HEp-2 cells (Fig. 5B). The majority (>90%) of NOD^mini^ and NOD^βTg^ mice tested had a speckled nuclear staining pattern, characteristic for SS development and found in parental NOD mice.

The onset of salivary gland dysfunction and presence of autoantibodies relies on B cells involvement and is dependent on IL-4 mediated IgM to IgG1 class switching [48]–[50]. Analysis of IgG1 concentration in sera of experimental mice revealed increased levels in older mice with the highest level in NOD^mini^ mice (Fig. 5C). This increase in IgG1 titer correlates with detection of auto-antibodies. Finally, to evaluate glandular dysfunction, we quantified secretion of saliva in 20 wks old mice. Both NOD^βTg^ and NOD^mini^ mice had reduced saliva flow as compared to healthy B6 mice and at the level of the reference parental NOD mice (Fig. 5D). Taken together, glandular infiltration, autoantibody production, and defective salivary secretion is diagnostic of Sjögren’s syndrome in transgenic NOD^βTg^ and NOD^mini^ mice, similarly to the parental strain of NOD mice. Interestingly, the timing of infiltrates and levels of autoantibodies and total IgG1 production vary between analyzed mice, which differ only by TCR repertoire diversity.

### Diversity of TCR repertoire on CD4^+^ T cells in NOD^mini^ mice

**Figure 6:**
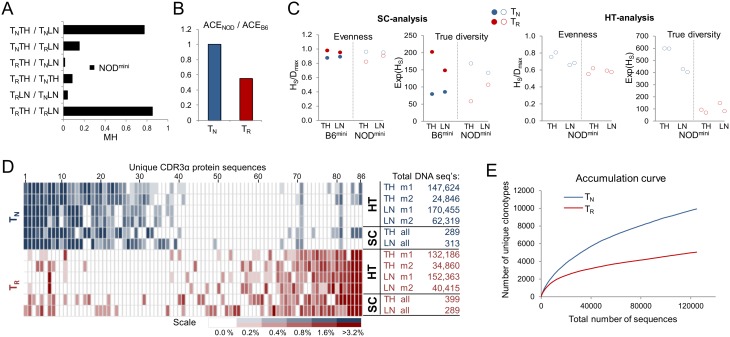
TCR repertoire of naïve and regulatory T cells in NOD^mini^ mice. (A) Similarity between indicated populations (first 289 single cell sequences per each population) was estimated based on Morisita-Horn index (MH). T_N_ - naïve CD4^+^Foxp3^−^CD45RB^+^CD62L^+^ T cells, T_R_ - regulatory CD4^+^Foxp3^+^ T cells, TH - thymus, LN - lymph nodes. (B) Ratio of richness of TCRs on T_N_ and T_R_ cells between NOD^mini^ and B6^mini^ mice. Abundance coverage estimator (ACE) was calculated based on 578 sequences for each population combined from thymus and peripheral lymph nodes. (C) Evenness and effective number of species (ENS) for analyzed TCR repertoires. Shannon evenness index was calculated as Shannon entropy (H_s_) divided by maximum diversity D_max_, where D_max_ equals natural logarithm of number of unique sequences in analyzed population. ENS (true diversity) was calculated as exponential of Shannon entropy. (D) Comparison of frequency of 20 most dominant unique protein CDR3 clonotypes found in each population indicated on the right of the heat map. Table indicates analyzed populations from lymph nodes and thymii by single cell analysis (SC) and high throughput sequencing (HT). Experimental mouse 1 and 2 are marked as m1 and m2. Numbers next to each population indicate total numbers of DNA sequences analyzed. All 86 unique CDR3α protein sequences in the heat map are shown in Table S1. (E) Accumulation curve of unique DNA clonotypes observed after accumulation of 124,730 sequences for T_N_ cells and 124,696 for T_R_ cells. (A–C) All indices were computed based on DNA sequences for each population using software SPADE and EstimateS8.2.

Lack of certain specificities in NOD^mini^ TCR repertoire and the previously reported lower TCR diversity of thymic Treg cells on NOD background prompted us to take a closer look at the similarity and diversity of TCRα^mini^ repertoires. To determine the influence of TCR diversity reduction on the selection efficiency of TCRs in NOD mice we started with a single cell analysis to compare to a previously analyzed similar model of TCR^mini^ transgenic mice on “healthy” C57BL/6 background (B6^mini^) [36]. We compared the similarity between TCR sequences from single cell sorted T_N_ (CD4^+^Foxp3^−^CD45RB^+^CD62L^+^) and Treg (CD4^+^Foxp3^+^) cells from thymii and peripheral lymph nodes of NOD^mini^ mice. As expected, based on the Morisita-Horn index, the highest similarity was observed between thymic and peripheral subsets of T_N_ or Treg cells, whereas comparison between populations of T_N_ and Treg cells showed mostly non-overlapping repertoires with values similar to those observed on B6 background (Fig. 6A and [36]). Previously we’ve shown that based on abundance coverage estimator (ACE), estimated richness (total unique CDR3α clonotypes in the population) of Treg cells in B6^mini^ mice significantly exceeded estimated richness of T_N_ cells [36]. Although ACE underestimates true richness at low sample size, it accounts for “unseen sequences” based on low abundance data and is suitable for comparative analyses. We combined thymic and peripheral sequences for each population and calculated the ACE index, based on 578 DNA sequences per subset. The ACE values for T_N_ and Treg cells were respectively 1187 and 994 for NOD^mini^ mice, and 1184 and 1815 for B6^mini^ mice. Interestingly, estimated ACE value for TCRs on T_N_ cells from NOD^mini^ mice was comparable to the ACE value for TCRs on T_N_ cells from B6^mini^ mice. However when we compared the ratio of ACE values for Treg cells between NOD^mini^ and B6^mini^ mice the number of possible unique TCRs on NOD background was reduced by almost 50% (Fig. 6B).

Previously it has been reported that based on analysis of two selected VJ (TCRα) or one VDJ (TVRβ) rearrangements in wild type mice, TCR repertoire of Treg cells in NOD mice was less diverse as compared to conventional T cells in the thymus, but also less diverse in the thymus of B6 mice [9]. These differences were observed based on calculation of Shannon entropy and normalization to the logarithm of unique sequences [9]. Such transformation is a measure of distribution of frequency of individual species and is a good measure of relative evenness of assemblage [51], [52]. Together with richness, evenness is a descriptive measure of diversity not the diversity *per se* [51]. Therefore, as explained by Jost, to put the estimates in perspective, we converted Shannon entropy (diversity index) to “true diversity” by calculating “numbers equivalent” also called “effective number of species” (ENS), to preserve linear scale of comparison [51], [53]. The ENS measure represents diversity of a particular sample, and the numeric value represents the theoretical number of equally common unique sequences in the assemblage. Comparison of “true diversity” between B6^mini^ and NOD^mini^ mice showed almost reversal of ratios of diversities between TCRs on T_N_ and Treg cells, with the differences more profound in the thymus than in the periphery (Fig. 6C). These differences in NOD^mini^ mice were confirmed by high throughput sequencing, and were consistent regardless of total numbers of sequences analyzed (Fig. 6C, D). Lower diversity of Treg TCR repertoire was visualized empirically by plotting accumulation curves of observed sequences from peripheral T_N_ and Treg cells (Fig. 6E). These curves show that accumulation of unique CDR3 regions from the first 120 thousands of sequences for each population gives twice as many unique DNA clonotypes in T_N_ (9757) as compared to Treg cells (4968). This ratio is reversed in comparison to accumulation curves observed in B6^mini^ mice [36]. Collectively our data show that although TCR diversity on Treg cells in NOD^mini^ mice is reduced, conventional T cells retain diverse TCR repertoire at least at the levels found on B6 background.

## Discussion

In this study we investigated the impact of the reduction of TCR diversity on the development of two autoimmune diseases in NOD mice; T1D and SS. Previously it has been shown that T1D can develop despite use of transgenic TCRβ chains or reduction of precursor frequency of potentially diabetogenic T cell clones. In our model overall diversity of TCRs was reduced by allelic exclusion caused by use of transgenic TCRβ chain that was not only pathogenically irrelevant, but also was originally selected in B6 mice on I-A^b^ molecule. Despite normal distribution of Vα families in NOD^βTg^ mice, neither insulitis nor diabetes developed. Conversely, these mice developed infiltrates in salivary and lacrimal glands, leading to autoantibody production and exocrine gland dysfunction. Further reduction of TCR diversity by generation of transgenic mice with TCR^mini^ repertoire, where one Vα segment is allowed to rearrange to only two Jα segments, did not prevent development of SS. Our results indicate that the difference between T1D and SS regarding the dependence on MHC polymorphism is directly correlated to the magnitude of possible TCR/peptide/MHCII interactions participating in the autoimmune phase of the disease.

We show that the lack of development of diabetes or even insulitis in our NOD^βTg^ or NOD^mini^ transgenic mice is due to lack of specificity to the key immunodominant insulin B:9–23 peptide, which is known to be instrumental for the onset of the T1D in NOD mice. The use of transgenic TCRVβ14 chain in our mice did not dramatically influence the ability of its binding to different TCRα chains, as T cells from NOD^βTg^ mice use all Vα families with frequencies found in NOD mice (Fig. 1). This includes efficient amplification of the Vα13 family which contains TRAV5D-4 chain (Vα13s1) that was shown to be sufficient to elicit anti-insulin autoimmunity without bias toward particular Vβ family of TCRβ chain partners [28], [54]. Moreover, previous studies show that T cells using Vβ14 family were found on T cells specific to insulin antigen, T cells expanding in pancreatic lymph nodes or in T cells infiltrating pancreatic islets, showing that the Vβ14 family is not negatively influencing the development of diabetogenic TCRs [55]–[58]. One cannot exclude the possibility that this particular transgenic TCRVβ14 chain may be unable to pair with appropriate TCRα chain, preventing the ability of the expressed αβTCR to recognize the B:9–23 peptide. This selective requirement for a TCRβ chain would reinforce our observation that development of SS is less dependent than development of T1D on overall TCR diversity and a particular peptide/MHC combination.

Development of T1D relies on different insulin B:9–23 register recognition, allowing escape of specific T cells due to register shifting [11]–[13]. The lack of peripheral recognition of insulin B:9–23 in our transgenic mice can also be due to the impact of the limited TCR repertoire. Reduction of overall TCR diversity can influence (reduce) the precursor frequency of DP thymocytes bearing potentially autoreactive TCRs, resulting in more efficient negative selection in the thymus of NOD mice. It has been shown that early expression of transgenic αβTCR, due to ERK1/2 defect on NOD background results in greater commitment of the DN thymocytes to αβ lineage “overcrowding” DP compartment [8]. Our TCRα^mini^ transgene has natural timing of expression, similar to B6^mini^ and polyclonal NOD mice, where pre-TCR signaling is not perturbed by early expression of the transgene [36]. As suggested by Mingueneau et. al., in the polyclonal repertoire on the NOD background, the ERK1/2 defect increases the affinity threshold of positive selection, shifting the selection window of thymocytes toward self-reactivity, however not impacting the efficiency of negative selection. This results in higher overall self-reactivity of peripheral effector T cells and possibly explains lower diversity of thymic Treg cells on NOD background [8], [9]. Considering partial overlap of specificities between Treg and autoreactive T cells, one could suggest similar effect of lower diversity on autoreactive population. However weak and unstable peptide-binding property of I-A^g7^ molecule does not favor the elimination or inactivation of autoreactive T cells [59]. This instability may have additional influence on “a leak” of autoreactive T cells, but also on inefficient generation of Treg population, which may require longer or stronger interactions with self MHC/peptide complexes [60], [61]. Therefore the shift in selection window will not impact autoreactive T cells as much as it will impact Treg cells, after all, Treg development relies on recognition of self-peptide/MHCII complexes in the thymus, whereas thymic escape of autoreactive T cells relies on avoidance of such complexes during negative selection. It is possible that in our model, we reached the threshold of diversity required to generate autoreactive TCR repertoire without “holes” in specificities. Therefore despite reduced TCR diversity of Treg cells mice do not develop diabetes and we were unable to detect insulin B:9–23 specific T cells in the periphery. Similarly, the comparison of TCR^mini^ repertoires between B6 and NOD backgrounds shows minimal impact of the NOD genotype on TCR diversity of conventional CD4^+^ T cells however it substantially reduces the TCR diversity on Treg cells in NOD mice.

Development of SS in NOD^mini^ mice is especially interesting, since CD4^+^ T cells are instrumental in immunopathogenesis and their recognition of self antigens is essential for the onset and progression of the disease [34], [62]. It shows that the TCR^mini^ repertoire is diverse enough not only to drive glandular infiltration and activation of the CD4^+^ T cells but also the repertoire is still diverse enough to support the full development of the disease with production of Th2-dependent IgG1 pathogenic autoantibodies (Fig. 5C) [50]. Moreover, we noticed differences in timing of infiltrates, levels of autoantibodies and total IgG1 production between transgenic mice and parental NOD mice, which indicates different frequencies of certain TCR specificities between mice. In congenic strains of NOD mice models of SS (NOD.B10-H2^b^, NOD.H2^p^, NOD.H2^q^, NOD.H2^h4^) replacement of I-A^g7^ with other MHC molecules does not prevent salivary and lacrimal gland infiltration and decreased saliva and tear production [16], [48], [63]. Interestingly, in NOD.H2^h4^, contrary to the parental NOD strain, there is a high frequency of ANA with a high proportion of SSA/Ro and SSB/La observed [63]. Also, NOD.H2^q^ mice exhibit increased production of lupus-like types of autoantibodies and develop nephritis, as compared to NOD and NOD.H2^p^ mice [16]. This weak dependence of SS on a particular MHC haplotype in NOD mice correlates with our data that show development of SS despite limited TCR diversity. In human studies it was suggested that production profiles of certain autoantibodies were associated with HLA-DR haplotypes rather than with clinical manifestations [64], however studies of familial inheritance in patients with SS showed a linkage between particular HLA and disease susceptibility [65], [66]. The most recent comprehensive analysis by Sjögren’s Genetics Network showed that HLA has the strongest linkage to the SS, although it is not on the level of T1D [17]. Certain HLA haplotypes will influence binding diversity of self or environmental peptides and the nature of antigen presentation to T cells during thymic development or during immune responses in the periphery. Our results from the mouse model emphasize that SS may be less affected by requirement of a unique key antigen/MHCII combination but rather may be more influenced by a wider range of overall TCR/peptide/MHC interactions involved in the onset/progression of the disease. It can be due to a combination of cross-reactivity of the TCR repertoire on SS-specific T cells, wider range of antigens presented by MHCII molecules, higher peripheral self-reactivity of effector T cells, increased tissue expression of MHCII complexes on salivary epithelial cells and de novo expression or post-translational modification of self-antigens [67]–[69].

## Supporting Information

Table S1 **Protein sequences of unique CDR3α regions from the heat map of** **Fig. 6D** **.**
(DOCX)
