# Supplementary material for: Reduction of T Cell Receptor Diversity in NOD Mice Prevents Development of Type 1 Diabetes but Not Sjögren’s Syndrome
Source: PLoS One. 2014 Nov 7;9(11):e112467. doi: 10.1371/journal.pone.0112467 (PMC4224485; doi:10.1371/journal.pone.0112467)
Supplement: Table S1 — Protein sequences of unique CDR3α regions from the heat map of Fig. 6D . (DOCX) [file pone.0112467.s001.docx]

**Table S1**

Protein sequences of unique CDR3α regions from the heat map of Fig. 6D.

| Pos. | Protein Sequence |
| --- | --- |
| 1 | CAASGNTGGLSGKLTFG |
| 2 | CAASAGSNYQLIWG |
| 3 | CAASANTGGLSGKLTFG |
| 4 | CAASLNTGGLSGKLTFG |
| 5 | CAASARSNYQLIWG |
| 6 | CAASGGLSGKLTFG |
| 7 | CAASGGDSNYQLIWG |
| 8 | CAASGDSNYQLIWG |
| 9 | CAASAHSNYQLIWG |
| 10 | CAASGGSNYQLIWG |
| 11 | CAAVDSNYQLIWG |
| 12 | CAARESNYQLIWG |
| 13 | CAASAWDSNYQLIWG |
| 14 | CAASARNYQLIWG |
| 15 | CAASQDSNYQLIWG |
| 16 | CAATDSNYQLIWG |
| 17 | CAASESNYQLIWG |
| 18 | CAANDSNYQLIWG |
| 19 | CAAIDSNYQLIWG |
| 20 | CAASRGSNYQLIWG |
| 21 | CAASTGNYQLIWG |
| 22 | CAAKDSNYQLIWG |
| 23 | CAALMDSNYQLIWG |
| 24 | CAASGMDSNYQLIWG |
| 25 | CAASGVDSNYQLIWG |
| 26 | CAASAGGLSGKLTFG |
| 27 | CAASPNYQLIWG |
| 28 | CAASDRSNYQLIWG |
| 29 | CAASEGGLSGKLTFG |
| 30 | CAASGKDSNYQLIWG |
| 31 | CAEDSNYQLIWG |
| 32 | CAASVGSNYQLIWG |
| 33 | CAASAGGSNYQLIWG |
| 34 | CAASRDYQLIWG |
| 35 | CAATQDSNYQLIWG |
| 36 | CAASGGNSNYQLIWG |
| 37 | CAARKSNYQLIWG |
| 38 | CAASEDSNYQLIWG |
| 39 | CAASVGSDYQLIWG |
| 40 | CAVVDSNYQLIWG |
| 41 | CAASEGYQLIWG |
| 42 | CAAEDTGGLSGKLTFG |
| 43 | CAASSNSNYQLIWG |
| 44 | CAARSAGGLSGKLTFG |
| 45 | CAASPGNYQLIWG |
| 46 | CAARGTGGLSGKLTFG |
| 47 | CAASGNSNYQLIWG |
| 48 | CAARSNYQLIWG |
| 49 | CAASKDSNYQLIWG |
| 50 | CAASGPGGLSGKLTFG |
| 51 | CAANSNYQLIWG |
| 52 | CAASAHNYQLIWG |
| 53 | CAATGSNYQLIWG |
| 54 | CAASPIMDSNYQLIWG |
| 55 | CAASPSSDSNYQLIWG |
| 56 | CAASGSGGLSGKLTFG |
| 57 | CAANWDSNYQLIWG |
| 58 | CAARRLSGKLTFG |
| 59 | CAGLDSNYQLIWG |
| 60 | CAAQDSNYQLIWG |
| 61 | CAANDGNYQLIWG |
| 62 | CAWTGGLSGKLTFG |
| 63 | CAASAKGNYQLIWG |
| 64 | CAASAPRGNYQLIWG |
| 65 | CAAAGGLSGKLTFG |
| 66 | CAVTGGLSGKLTFG |
| 67 | CAAISNYQLIWG |
| 68 | CAASGETGGLSGKLTFG |
| 69 | CEGTGGLSGKLTFG |
| 70 | CAASWGNYQLIWG |
| 71 | CAARDSNYQLIWG |
| 72 | CAAIYSNYQLIWG |
| 73 | CAASASNYQLIWG |
| 74 | CAASADYQLIWG |
| 75 | CAASGSNYQLIWG |
| 76 | CAASEVSNYQLIWG |
| 77 | CAAMDSNYQLIWG |
| 78 | CAGTGGLSGKLTFG |
| 79 | CAASDYSNYQLIWG |
| 80 | CAASDSNYQLIWG |
| 81 | CAATGGLSGKLTFG |
| 82 | CAATGDSNYQLIWG |
| 83 | CARMDSNYQLIWG |
| 84 | CAALDSNYQLIWG |
| 85 | CAASNSNYQLIWG |
| 86 | CAASGTGGLSGKLTFG |
